# To boost or to CRUNCH? Effect of effortful encoding on episodic memory in older adults is dependent on executive functioning

**DOI:** 10.1371/journal.pone.0174217

**Published:** 2017-03-22

**Authors:** Li Fu, Joseph H. R. Maes, Roy P. C. Kessels, Sander M. Daselaar

**Affiliations:** 1 Donders Institute for Brain, Cognition and Behaviour, Radboud University, Nijmegen, The Netherlands; 2 Department of Medical Psychology, Radboud University Medical Center, Nijmegen, The Netherlands; University of Akron, UNITED STATES

## Abstract

It is essential to develop effective interventions aimed at ameliorating age-related cognitive decline. Previous studies found that effortful encoding benefits episodic memory in older adults. However, to date it is unclear whether this benefit is different for individuals with strong versus weak executive functioning (EF). Fifty-one older adults were recruited and divided into low (N = 26) and high (N = 25) functioning groups, based on their EF capacity. All participants performed a semantic and a perceptual incidental encoding task. Each encoding task was performed under four difficulty levels to establish different effort levels. Encoding was followed by a recognition task. Results showed that the high EF group benefitted from increased effort in both tasks. However, the low EF group only showed a beneficial effect under low levels of effort. Results are consistent with the Compensation-Related Utilization of Neural Circuits Hypothesis (CRUNCH) and suggest that future research directed at developing efficient memory strategies to reduce negative cognitive aging effects should take individual cognitive differences among older adults into account, such as differences in EF.

## Introduction

In the past few decades, an increasing number of studies have focused on cognitive aging and factors that could counteract adverse aging effects. Several studies have shown that environmental support, for example in the form of providing mnemonic strategies, can alleviate cognitive-aging induced episodic memory decline [1, 2]. One type of environmental support in a broader sense consists of manipulating task demands to enhance depth of processing of the to-be-learned material, such as in tasks that encourage deep semantic encoding [3–5]. In a recent study [6] comparing young (YAs) and older adults (OAs), we found that encoding especially enhances later memory retrieval in OAs if encoding takes place under relatively demanding conditions that implicate much cognitive effort. This effect is in accordance with the environmental-compensation view [6–8], which states that high task demands may encourage OAs to use their limited cognitive resources in a more efficient way, thereby compensating for their cognitive deficits. However, the amount of cognitive effort invested in a task, which later benefits episodic memory retrieval, might be highly dependent on the individual’s cognitive capacities.

In principle, two lines of reasoning can be adopted regarding the question of who will benefit most from effortful semantic encoding. The first hypothesis is that OAs who have pronounced cognitive deficits will profit more from this type of environmental support than individuals with relatively intact cognitive abilities. This hypothesis is based on the idea that there is more room for a compensation-based improvement in the former than the latter group of individuals. As shown by Clark et al. [9], elderly with lower education, presumably having a relatively low cognitive reserve, take more benefit from training speed of processing compared to those with higher levels of education. Other supporting evidence is provided by a recent study showing that, compared to young adults, children displayed more positive transfer effects of a cognitive training program, which might also be due to their lower cognitive capacity [10].

An alternative hypothesis is that larger benefits can be expected for individuals with relatively intact as opposed to poor cognitive abilities. This hypothesis relates to the Compensation-Related Utilization of Neural Circuits Hypothesis (CRUNCH) [11]. According to this model, OAs may reach a memory performance that is comparable to that displayed by YAs by recruiting more (also: bilateral as opposed to unilateral) prefrontal activation than shown by the YA [12, 13]. However, importantly, especially in OAs with relatively poor cognitive capacity, the cognitive compensatory mechanisms that can be mobilized is limited. It has been shown in working memory studies that, beyond a critical threshold, increasing cognitive demands results in a decreased activation of prefrontal brain areas and corresponding decreased performance (e.g., [14–17]). Hence, this model would suggest that individuals with relatively strong cognitive abilities will reach the critical threshold (i.e., display the CRUNCH effect) later compared to those with poor cognitive abilities. This suggests that the better functioning individuals will show a larger benefit of effortful encoding conditions, as compared to the lower functioning individuals.

Previous studies report a remarkable variability in cognitive capacity at old ages [18–21]. For example, some OAs exhibit severe memory impairments, whereas others are able to maintain a high level of functioning [22, 23]. However, variability at old age is especially present for one particular class of cognitive processes, namely executive functioning (EF). As a higher-level cognitive process, EF supervises and controls a wide range of more basic cognitive processes, thereby enabling goal-directed operations, such as the inhibition of task-irrelevant information, the implementation of strategies, the switching between tasks, the adjustment of behavior based on feedback, and planning [24, 25]. The executive functioning decline hypothesis is one of the main theories in the field of cognitive aging [26–28]. It postulates that EF decline is a hallmark of cognitive aging and may be the main mediator of age differences in cognitive capacities, especially in episodic memory. The EF decline hypothesis has been found particularly advantageous to explain episodic memory deficits during aging [29]. EF is engaged in conscious and strategic aspects of memory performance and the influence of EF on age-related memory differences appears especially prominent in resource-dependent and strategic memory conditions [28, 30, 31].

A previous study by Angel et al. [24] suggests that high EF may underlie the individual’s cognitive reserve capacity, by helping OAs implement efficient strategies as well as making use of environmental support, and consequently maintain a high level of episodic memory performance. In the present study, we aimed to more directly assess the effect of environmental support on OAs’ episodic memory performance as a function of differences in EF. Specifically, we applied EF as an index of individual differences in cognitive functioning and assessed whether OAs with low EF would benefit more from increasing cognitive effort in encoding than those with high EF, or vice versa.

In sum, in the current study, we aimed to answer the question concerning *who* benefits more from effortful encoding: OAs with low EF capacities, who have more deficits to compensate for, or OAs with high EF capacities, who can still make use of the provided environmental support. Several neuropsychological tests were applied to assess EF of OAs. High and low EF groups were formed based on their EF scores. We employed both incidental encoding tasks as used by Fu et al. [6]: a deep encoding task based on semantic relatedness between words and a shallow encoding task based on word size. Cognitive effort was manipulated by varying decision-making demands in both encoding tasks. Episodic memory performance, indexed by d-prime, was later tested using a recognition task. As shown previously [6], in general, effortful encoding benefits memory performance after both levels of processing (LoP: deep and shallow encoding). If indeed one group benefits more from effortful encoding than another, this differential effect should hold for both encoding tasks.

## Materials and methods

### Participants

Fifty-one OAs, aged 60 to 80 years (M = 66.96, SD = 5.50, 20 women), were recruited by an advertisement in local newspapers. All participants were native Dutch speakers without a history of neurological or psychiatric illnesses (self-report) and scored higher than 25 on the Mini Mental State Examination (MMSE, [32]; M = 29.29, SD = 0.88, range = 26–30). The group of participants were divided into a low and a high EF group using a median split based on the composite z-score of neuropsychological tests (see Materials below). The groups did not differ in age and education. The high EF group displayed significantly higher scores on the MMSE, the Backward Digit Span task, and the Mental Arithmetic task (see Table 1) than the low EF group. All participants signed an informed consent form and received €40 as remuneration. The study was approved by the Ethics Committee of the Faculty of Social Sciences of the Radboud University and all experimental manipulations were performed in accordance with the approved guidelines and the declaration of Helsinki.

**Table 1:** Demographics and neuropsychological results of the two groups.

	Low-EF	High-EF	t (df = 49)	*p* (2-tailed)
**N**	26	25		
**Gender (female)**	15	5		
**Age**	67.23 (5.69)	66.68 (5.40)	0.354	.725
Education*	5.58 (1.21)	6.00 (0.91)		.202
**MMSE**	29.04 (1.04)	29.56 (0.58)	-2.199	.033
**Backward Digit Span**	9.31 (2.11)	13.48 (2.80)	-8.359	.000
**Mental Arithmetic**	9.85 (1.97)	14.68 (2.16)	-6.022	.000

*Education level was calculated based on the Dutch educational system using a 7 point scale, with 1 = less than primary education and 7 = academic degree [33]. The comparison between two groups was conducted using a Mann-Whitney U test.

### Materials

The materials used in this study were the same as described in Fu et al. [6] except for the neuropsychological tests. Detailed methods are described below.

#### Deep encoding task

**Fig 1:**
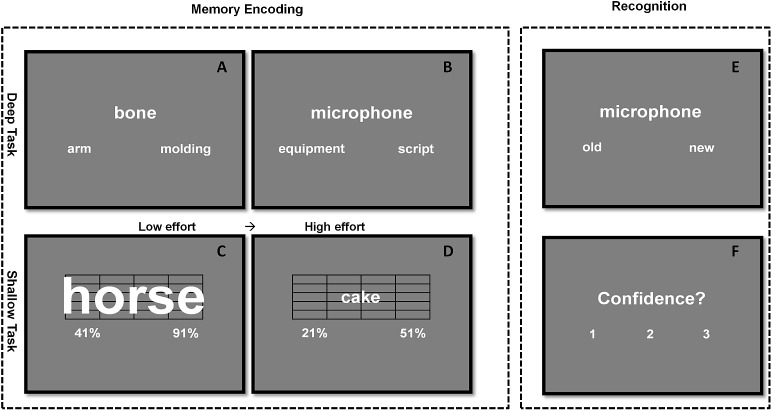
Design of the experiment. Example of encoding decision-making trials in easy (A, C) and difficult (B, D) deep and shallow encoding tasks, respectively. In the deep encoding task (A, B), participants indicate which of the two bottom words is more semantically related to the target word at the top. In the shallow task (C, D), participants choose a value representing the correct percentage of the height of the grid occupied by the word. Sample recognition memory task: participants indicate whether a word is old (appeared in the previous encoding task) or new (E), together with how confident they are (on a scale from one to three) about the choice (F).

The deep encoding task comprised of 360 trials, each beginning with a fixation cross (500 ms), followed by a word triplet. Participants were asked to indicate which of the two words displayed at the bottom of the screen was more semantically related to the target word at the top by pressing corresponding buttons on the keyboard. All words and their relatedness scores were retrieved from the LSA database (lsa.colorado.edu) and matched on word length and frequency for each difficulty level. Trials proceeded in a self-paced fashion with a 5-s response limit. For the purpose of promoting variations in semantic cognitive effort, each triplet was categorised to one of four difficulty levels determined by the difference between the semantic relatedness score of each top-bottom pair. These difference levels were set at 0.30 (easy, Fig 1A), 0.20, 0.10, or 0.05 (hard, Fig 1B). The smaller the difference between two scores, the more cognitive effort was assumed to be required to make the ‘encoding’ decision. The resulting 360 triplets were translated from English to Dutch by five independent native Dutch speakers, and presented randomly to each participant.

#### Shallow encoding task

The shallow encoding task also contained 360 trials. On each trial, a target word was overlaid on a rectangular grid, with two percentage values presented below. Participants were instructed to choose a value representing the correct percentage of the height of the grid occupied by the word. Similar to the deep encoding task, four difficulty levels were set to modulate perceptual cognitive effort. These levels were defined by the differences between the bottom two values, 90%, 70%, 50% (Fig 1C), and 30% (Fig 1D). Since it was necessary that participants actually read the word while making the size judgement for encoding to take place, 36 pseudo-word trials were added as fillers. Participants could receive an extra bonus for skipping each filler by pressing the space key.

#### Recognition memory task

The 720 target words from the deep and shallow encoding tasks were intermixed with 360 new words and presented in random order during the “old/new” recognition task. Besides an old or new judgement, participants were also asked to make a confidence judgement on a scale from one to three: 1) “guess”, 2) “probably”, and 3) “definitely”. However, the latter data are not relevant for the present study and will not be reported or discussed. Similar to the encoding tasks, recognition trials proceeded in a self-paced fashion with a 5-s response limit, with short breaks after every 270 trials (see Fig 1E & 1F).

#### Neuropsychological tests

Similar to the study by Glisky and her colleagues [34], three neuropsychological tests were used to measure EF: 1) Backward Digit Span from the Wechsler Adult Intelligence Scale–Fourth Edition (WAIS-IV)[35], 2) Mental Arithmetic, and 3) Mental Control from the Wechsler Memory Scale–Fourth Edition (WMS-IV)[36], and two neuropsychological tests were used to evaluate MB (MB: memory binding function, see S1 File for details): 1) Logical Memory, and 2) Word pairs Association from the WMS-IV. However, we did not use participant’s performance on the Mental Control test for determining the composite EF score due to a ceiling effect (Full score = 8, Median = 8, Mean = 7.82, SD = .62). Participants’ performance on each of the other two tests of EF was computed by norm scores that later was converted to a z score. Subsequently, the average of the two z scores was taken as the composite EF score, representing each individual’s EF capacity. The composite MB score was calculated in the same fashion as the composite EF score.

### Procedure

Participants visited the research lab at Radboud University twice for this study. Neuropsychological tests were administered at the first appointment, together with the MMSE. Participants also practiced the deep and shallow encoding tasks at this session. The actual experiment was conducted during the second session, which took place 2–5 days after the first appointment. The experiment comprised two blocks of the deep encoding task followed by two blocks of the shallow encoding task with a 3-min break in between. The recognition task consisted of the four blocks in reverse order to counteract possible floor effects of shallow encoding, which started immediately after participants finished the encoding tasks. Before starting the actual experiment, participants acquainted themselves again with the task by instructions. The experiment was designed using PsychoPy [37].

### Data analysis

The data from the two encoding blocks were collapsed for each task. Words that were not responded to during encoding, had a response time (RT) ± 3SD away from the total sample’s mean, or with an RT < 200 ms were removed from later calculation of d-primes. Applied cognitive effort was measured by RTs. For each encoding task and participant, four levels of cognitive effort were established using rank-based percentile cuts: Level 1: RT < 25%ile; Level 2: 25%ile ≤ RT< 50%ile, Level 3: 50%ile ≤ RT < 75%ile; and Level 4: RT≥ 75%ile. For the low EF group, the RT in the deep encoding condition ranged from 1739 ms at effort level 1 to 3179 ms at effort level 4. For the high EF group the corresponding values were 1867 and 3311 ms. For the shallow encoding condition, the RT for the low EF group ranged from 1320 ms at effort level 1 to 2727 ms at effort level 4. For the high EF group the corresponding values were 1365 and 3049 ms. A Group × Encoding condition × Cognitive effort analysis of variance using these RT data failed to reveal a significant effect involving the group factor.

Memory performance at each cognitive effort level for both deep and shallow encoding tasks was calculated by d-prime [38]. A repeated-measures generalized linear model (GLM) analyses of variance was conducted with d-prime as the dependent variable, Group (High EF vs. Low EF) as between-subject factor, LoP (deep vs. shallow) and Cognitive effort (4 levels) as within-subject factors. Post-hoc tests were conducted to examine significant interaction effects. All statistical tests employed *p* < .05 as criterion for significance and effect sizes (partial eta-squared) are reported.

## Results

### Main effects

The repeated-measures GLM revealed three main effects: 1) LoP, *F*(1, 49) = 21.81, *p* < .001, *η*_p_^2^ = .31, reflecting higher d-primes after deep (*M* = .53 *SD* = .29 than shallow (*M* = .33, *SD* = .16 encoding; 2) Group, *F*(1, 49) = 5.92, *p* = .019, *η*_p_^2^ = .11, reflecting higher d-primes in the high EF group (*M* = .49 *SD* = .17) than low EF group (*M* = .37 *SD* = .17); and 3) Cognitive effort, *F*(3, 47) = 21.79, *p* < .001, *η*_p_^2^ = .31. A post-hoc test with Bonferroni-correction for multiple comparisons revealed that the latter effect reflected differences in recognition memory performance at levels 1 (*M* = .32, *SD* = .20) and 2 (*M* = .43, *SD* = .19) (*p* < .001), 1 and 3 (*M* = .49, *SD* = .20) (*p* < .001), and 1 and 4 (*M* = .48 *SD* = .19) (*p* < .001), indicating that memory recognition increased with more cognitive effort devoted to memory encoding.

### Interactions between Group, Effort, and LoP

**Fig 2:**
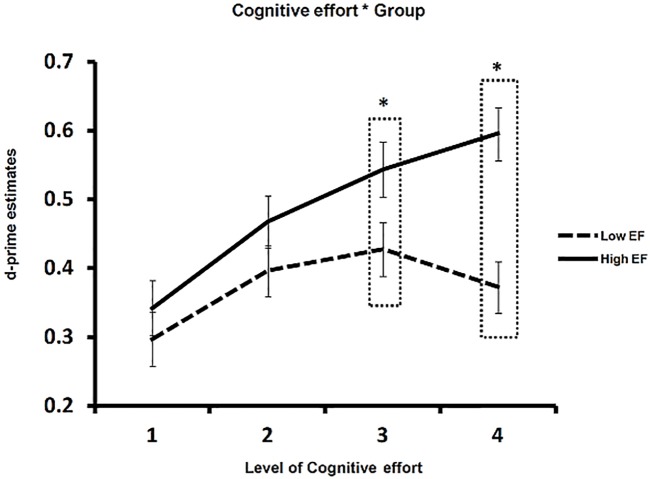
Illustration of CRUNCH effect. A difference in memory performance between low and high EF groups appeared at cognitive levels 3 and 4. A significant quadratic component appeared for the low EF group indicating a CRUNCH effect.

Of primary interest, the interaction between Group and Cognitive effort was significant, F(3, 147) = 5.51, p = .001, ηp2 = .11, reflecting a significant better performance for the high compared to low EF group at Cognitive level 3 (p = .046) and 4 (p < .001) (see Fig 2). Also, a separate ANOVA with effort as single factor for each group separately revealed a significant quadratic component (p = .010, ηp2 = .24), suggestive of a CRUNCH effect, but no linear component (p = .079, ηp2 = .12) for the low EF OAs. Pairwise comparisons revealed that the quadratic effect represents a significant difference in memory performance between levels 1 and 3 (p = .035). For the high EF group, only the linear component was highly significant (p <.001, ηp2 = .81) and pairwise comparisons showed that, apart from levels 3 and 4 (p = .243), the difference in memory performance between all other pairs was significant (ps <.05). There was no three-way interaction among Group, LoP, and Cognitive effort (F <1).

Although we did not observe a three-way interaction between Group, LoP, and Cognitive effort, we performed a Group × Cognitive effort GLM for deep and shallow tasks separately to see if the effect of EF difference holds similarly for both tasks.

**Fig 3:**
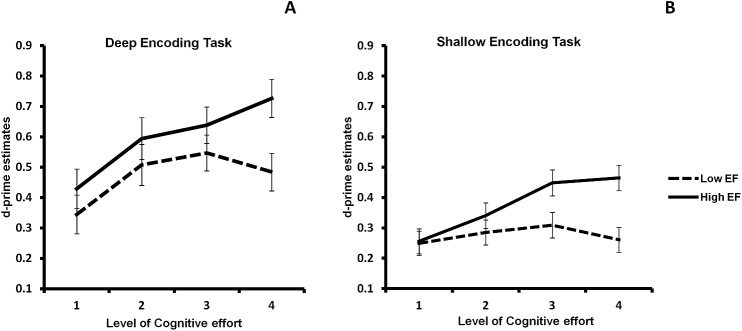
Effect of cognitive effort in the deep and shallow encoding conditions for each of the two groups. When viewing separately, the CRUNCH effect was present in the deep encoding condition in the low EF group (A), whereas this group did not benefit from encoding effort in the shallow encoding task (B).

For the deep encoding task, the GLM revealed a significant main effect of Cognitive effort, *F*(3, 47) = 17.04, *p* < .001, *η*_*p*_^2^ = .26, and a marginally significant interaction effect between Group and Cognitive effort, *F*(3, 147) = 2.52, *p* = .06, *η*_*p*_^2^ = .05 (Fig 3). Subsequent simple main effect analysis showed that there was no difference in d-prime between the two groups for effort levels 1−3, but importantly, there was a highly significant difference at the highest level of cognitive effort, t(49) = -2.74, *p* = .009. Moreover, polynomial contrasts showed a quadratic component in the low EF group (*p* = .007) together with a linear component (*p* = .021), whereas there was only a linear component in the high EF group (*p* < .001; quadratic component: *p* = .159). This provides support for the CRUNCH model in the low EF group (Fig 3A). For the shallow task, the Group × Cognitive effort GLM revealed a main effect of Cognitive effort, *F*(3, 47) = 6.64, *p* < .001, *η*_*p*_^2^ = .12, a main effect of Group *F*(1, 49) = 5.22, *p* = .027, *η*_*p*_^2^ = .10, and an interaction effect between Group and Cognitive effort. Further analysis showed that a difference in memory performance between the two groups was present at cognitive effort level 3 (t(49) = -2.32, p = .025) and 4 (t(49) = -3.95, p = .001). Moreover, only the high EF group seemed to have benefited from increasing cognitive effort, *F*(3, 72) = 14.51, *p* < .001, *η*_*p*_^2^ = .38, whereas the low EF group did not, *F*(3, 75) = .56, *p* = .644, *η*_*p*_^2^ = .02 (see Fig 3B).

## Discussion

In this study, employing the same approach as in Fu et al. [6], we examined whose episodic memory will benefit most from increasing cognitive effort during encoding, OAs with low (low EF OAs), or those with high (high EF OAs), executive function capacity. The results revealed that, with more effort employed during encoding, recognition memory improved in high EF OAs. However, the beneficial effect appeared in the low EF group as a clear CRUNCH effect. Below, we discuss our main findings in separate sections.

### More room more improvement or less capacity earlier CRUNCH

In answering the question: who benefit most from scaffolded cognitive effort as environmental support for episodic memory enhancement, we tested two possible hypotheses supported by two different lines of reasoning. The first hypothesis stated that OAs who have pronounced cognitive deficits (in this study: the low EF group) will benefit more than individuals with relatively intact cognitive abilities (high EF group in this study). This hypothesis is based on the idea that there is more room for a compensation-based improvement in the former than the latter group of individuals. An alternative hypothesis is that larger benefits can be expected for individuals with relatively intact as opposed to poor cognitive abilities, which is derived from the CRUNCH model. This model is based on neuroimaging studies of working memory, and proposes that cognitive compensation mechanisms that can be mobilized is limited, and OAs with stronger cognitive capacities (high EF group) reach the limitation for these mechanisms (i.e. display the CRUNCH effect) later than OAs with weaker cognitive abilities (low EF group).

The current results support the latter hypothesis. Consistent with previous findings [6, 24], OAs with relatively strong EF capacities are able to make use of the advantage provided, by increasing effort during encoding, to support later retrieval from episodic memory. Critically, older people with relatively poor EF abilities indeed showed a CRUNCH effect. Although memory performance was improved by more cognitive effort at levels 2 and 3, no more improvement occurred at level 4, presumably due to the high processing requirement of the task that went beyond the individual’s capacity. Instead, the high EF OAs did not reach this crucial CRUNCH point with the current effort level settings, and therefore these individuals could continuously benefit from increasing effort in memory encoding. This could also explain the result that differences in episodic memory performance between high and low EF OAs only appeared at effort levels 3 and 4. To our knowledge, this is the first study that–adding to the previous *working memory* studies [11, 16, 17]–reports a CRUNCH effect in an *episodic memory* task with increasing task demands during encoding. It is quite likely that it can also be found in other cognitive domains, but that remains a question for future research.

### The importance of individual differences in aging research and therapeutic measures

In the past, cognitive aging has primarily been treated as a population-level phenomenon. Our data indicate that investigating individual differences in the cognitive aging process might be important, especially for the purpose of developing effective interventions. There are large differences in the magnitude of aging effects as a function of which specific aspect of cognitive functioning is studied [22, 23, 39]. Moreover, inconsistent results have been reported on the effectiveness of cognitive interventions developed to counter aging deficits on a group level [39–41]. More detailed knowledge about individual differences in aging-related changes in different cognitive abilities will be required to determine factors that contribute to these mixed results [42]. As shown in this study, the enhancement of episodic memory by environmental support, provided during encoding, is highly dependent on the remaining cognitive resources of the older adult. Although OAs with low EF could still benefit from increasing effort at relatively low task-demand levels of the deep encoding task, they could not benefit from effortful encoding at all in the shallow encoding task. Although including or excluding the MB score in the analyses did not significantly affect the results (see S1 File), we suggest that this lack of benefit for the low EF OAs might be due to the fact that the age-related memory decline is associated with MB deficits. Indeed, we observed a moderate positive correlation between EF and MB (r = .29, p < .05), similarly as Daselaar et al. [43] found. Accordingly, OAs with low EF also suffer from a relatively low MB, and we hypothesize that they could not automatically form meaningful memory traces in the shallow encoding task, since the instruction of the task directed attentional resources to process shallow/perceptual aspects of the stimuli. Instead, high EF elderly could still internally initiate semantic memory processing during the shallow encoding task since their MB function is relatively intact. Accordingly, this would implicate that possible interventions for OAs should take both EF and MB capacity into consideration for the purpose of developing proper training materials for counteracting their age-related memory decline. More generally, the present findings and considerations fit the conclusion derived from previous work that various task- and person-related parameters play an importantly role in finding or not finding age differences in the effect of environmental support [5, 44].

### Limitations of the present study and future directions

The current study had a few limitations. First, a median split approach was used to determine whether a participant belonged to the high or low EF group. Since most of our participants were highly educated, our study sample may not be representative of the general aging population. Future research should include more diverse populations, that is, also include older adults with lower education levels. Second, many studies suggest EF has different components such as inhibition, updating, and task switching. The EF tests we employed in this study might not cover all aspects of executive functioning. While our selection of tasks was based on the work of Glisky et al. [34], who confirmed that these tests all contribute to the EF factor by factor analysis, future studies may also want to include other aspects of EF. Third, the CRUNCH model we used in this study was based on results of neuroimaging studies. Although we observed a CRUNCH-like trend in our behavioral results, it is unclear whether this phenomenon is indeed the same as that observed in neuroimaging studies. Future studies could employ fMRI to investigate the influence of effortful encoding on increasing memory performance in OAs.

## Conclusions

Extending our previous study [6], the current study addressed the question whose memory performance benefits more from effortful encoding, elderly with low or high EF. Results revealed that high EF OAs, putatively possessing optimal cognitive reserve, can take advantage of environmental support in the form of promoting cognitive effort. However, older individuals with low EF could only benefit from this support in a limited way. Our findings support the CRUNCH model and underscore the importance of individual differences in aging research.

## Supporting information

S1 File MB as covariate. Using memory binding (MB) function as a covariate in measuring the influence of EF on episodic memory.
(PDF)

S2 File Data. Original data set of all measurements mentioned in this manuscript.
(XLSX)
