# Supplementary material for: To boost or to CRUNCH? Effect of effortful encoding on episodic memory in older adults is dependent on executive functioning
Source: PLoS One. 2017 Mar 22;12(3):e0174217. doi: 10.1371/journal.pone.0174217 (PMC5362088; doi:10.1371/journal.pone.0174217)
Supplement: S1 File — Using memory binding (MB) function as a covariate in measuring the influence of EF on episodic memory. (PDF) [file pone.0174217.s001.pdf]

## MB as covariate

Apart from EF, memory binding (MB) is another major function that should be considered in the context of individual differences in episodic memory aging. The MB-deficit hypothesis postulates that age-related memory deficits are the result of difficulties in binding features that constitute a coherent representation of episodic memory [1]. Evidence from both behavioral and neuroimaging studies suggest age-related deficits in both EF and MB, which are assumed to be two independent paths leading to episodic memory decline [2-5]. However, studies have reported a positive correlation between EF and MB [6]. To control for individual differences in MB, in this study, we included neuropsychological tests for MB, the score of which was initially applied as a covariate in the statistical analyses. However, including MB as covariate did not yield significant main or interaction effects involving this factor, and the results did not change when excluding MB as covariate. Therefore, MB was not included as part of the reported analyses.

## References


1. Naveh-Benjamin M. Adult age differences in memory performance: tests of an associative deficit hypothesis. J Exp Psychol Learn Mem Cogn 2000; 26:1170–87. PMID: 11009251

2. Daselaar S, Cabeza R. Age-related decline in working memory and episodic memory: contributions of the prefrontal cortex and medial temporal lobes. In: Ochsne K, Kosslyn SM, editors. The oxford handbook of cognitive

neuroscience, volume 1: core topics. New York: Oxford Univeristy Press; 2013. pp. 456–472.

3 Old SR, Naveh-Benjamin M. Differential effects of age on item and associative measures of memory: a meta-analysis. Psychol Aging 2008; 23:104–18. PMID: 18361660

4. Tromp D, Dufour A, Lithfous S, Pebayle T, Després O. Episodic memory in normal aging and Alzheimer disease: insights from imaging and behavioral studies. Ageing Res Rev 2015; 24:232-62. PMID: 26318058

5. van Geldorp B, Parra MA, Kessels RPC. Cognitive and neuropsychological underpinnings of relational and conjunctive working memory binding across age. Memory 2015; 23:1112–22. PMID: 25216357

6. Daselaar SM, Iyengar V, Davis SW, Eklund K, Hayes SM, Cabeza RE, et al. Less wiring, more firing: low-performing older adults compensate for impaired white matter with greater neural activity. Cereb Cortex 2015; 25:983-90. PMID: 24152545
